# Allele-specific repression of *Sox2* through the long non-coding RNA *Sox2ot*

**DOI:** 10.1038/s41598-017-18649-4

**Published:** 2018-01-10

**Authors:** Tobias C. Messemaker, Selina M. van Leeuwen, Patrick R. van den Berg, Anke E. J. ‘t Jong, Robert-Jan Palstra, Rob C. Hoeben, Stefan Semrau, Harald M. M. Mikkers

**Affiliations:** 1 Department of Molecular Cell Biology, Leiden University Medical Center, PO Box 9600, 2300RC Leiden, The Netherlands; 2 Department of Rheumatology, Leiden University Medical Center, PO Box 9600, 2300RC Leiden, The Netherlands; 3 Leiden Institute of Physics, Leiden University, 2333 RA Leiden, The Netherlands; 4 Department of Biochemistry, Erasmus University Medical Center, Ee634, 3000CA Rotterdam, The Netherlands

## Abstract

The transcription factor Sox2 controls the fate of pluripotent stem cells and neural stem cells. This gatekeeper function requires well-regulated Sox2 levels. We postulated that *Sox2* regulation is partially controlled by the *Sox2* overlapping long non-coding RNA (lncRNA) gene *Sox2ot*. Here we show that the RNA levels of *Sox2ot* and *Sox2* are inversely correlated during neural differentiation of mouse embryonic stem cells (ESCs). Through allele-specific enhanced transcription of *Sox2ot* in mouse *Sox2eGFP* knockin ESCs we demonstrate that increased *Sox2ot* transcriptional activity reduces *Sox2* RNA levels in an allele-specific manner. Enhanced *Sox2ot* transcription, yielding lower *Sox2* RNA levels, correlates with a decreased chromatin interaction of the upstream regulatory sequence of *Sox2* and the ESC-specific *Sox2* super enhancer. Our study indicates that, in addition to previously reported in trans mechanisms, *Sox2ot* can regulate *Sox2* by an allele-specific mechanism, in particular during development.

## Introduction

Correct gene regulation, which relies on the temporally and spatially controlled expression of lineage specific transcription factors, determines the success of development. Sox2 is such a transcription factor key to development. *Sox2* belongs to the family of high mobility group (HMG) DNA binding domain genes related to the sex determining gene Y (Sry) and together with *Sox1* and *Sox3*, *Sox2* forms the SoxB1 family. Sox2 exerts its cell type specific function by interaction with other homeodomain transcription factors, the POU domain protein Oct4, or the paired domain protein Pax6^1^. An important function of Sox2 is maintaining the stem cell state of either naïve or primed pluripotent stem cells^2^. Reduction or overexpression of Sox2 in mouse and human embryonic stem cells (ESCs) induces the differentiation into primarily endoderm and trophoectoderm-like cells, respectively^3–8^. Endogenous Sox2 levels also influence the germ layer fate of pluripotent stem cells. High endogenous levels steer pluripotent cells into the (neural) ectodermal lineage, whereas low levels promote mesendodermal differentiation^9^. Sox2 fulfills a similar role in neural stem cells (NSCs) in *vitro* and in *vivo*. Overexpression of Sox2 in NSCs of the developing spinal cord represses differentiation by counteracting transcription factor driven proneural programs, whereas Sox2 protein inhibition enhances differentiation^10,11^. In the developing eye, retinal progenitor cells lose their proliferation and differentiation capacity after *Sox2* ablation^12^. Reduced Sox2 levels (<40%) cause microphthalmia due to aberrant differentiation of the progenitor cells^12^. In addition, misexpression of Sox2 in astrocytes converts them into neuroblasts^13^, whereas it activates neural transcription programs in cells of mesodermal origin^14,15^. Thus, well-controlled and tightly-timed Sox2 activity appears to be important for correct neural development.

Sox2 activity is controlled by post-translational modifications, such as serine- and threonine phosphorylation, sumoylation, ubiquitination, and acytelation^16^. These modifications affect localization, DNA binding and stability. However, Sox2 activity is to a great extent controlled at the transcriptional level. The requirement for well-balanced, tightly controlled, and cell type specific expression explains the complex genomic architecture of the *Sox2* locus. Multiple enhancer elements that drive tissue specific expression have been identified in the 200 kb region surrounding *Sox2* ^17–20^. Consequently, endogenous expression has only been fully recapitulated in transgenic mice through a knockin approach where one of the *Sox2* alleles was replaced by a marker gene^12,21,22^ or through introduction of bacterial artificial chromosomes (BACs) containing >200 kb of *Sox2* genomic sequences^23^.

Protein encoding genes like transcription factors and chromatin modifiers are key to transcription activation. However, RNA genes that do not encode proteins can fulfill transcriptional regulatory roles as well. Long non-coding RNAs (lncRNAs), which are >200 nucleotides in length, seem to have in particular evolved for controlling genes at a transcriptional level^24^. LncRNA-mediated transcription regulation is instructed in cis or in trans. Allele-specific in cis mechanisms include recruitment of chromatin modifying complexes repressing transcription^25^ or activating transcription^26^, transcriptional interference preventing transcription factor access^27,28^, or gene looping^29^. Recently, a lncRNA gene called *Sox2* overlapping transcript (*Sox2ot*) that is transcribed in the same direction as *Sox2* and is polyadenylated downstream of *Sox2* was described^30,31^. To date several studies investigating the function of *Sox2ot* have been reported^32–34^. These studies utilized knockdown or overexpression of *Sox2ot* in cancer cell lines and the results have indicated a role of S*ox2ot* in regulating proliferation as well as regulating *Sox2*. *Sox2ot* levels were invariably positively correlated with *Sox2*, however, the underlying regulatory mechanism has remained unknown.

In this study we evaluated expression of *Sox2ot* during development and studied the effect of *Sox2ot* overexpression in modified mouse ESCs that allow discrimination between cis and trans regulatory effects. On basis of our data we propose that during development *Sox2ot* expression is mainly restricted to neural cell types and that, in contrast to previous reports, enhanced *Sox2ot* transcriptional activity negatively affects *Sox2* RNA levels in an allele-specific manner.

## Results

### Characterization and conservation of *Sox2ot* transcripts

**Figure 1:**
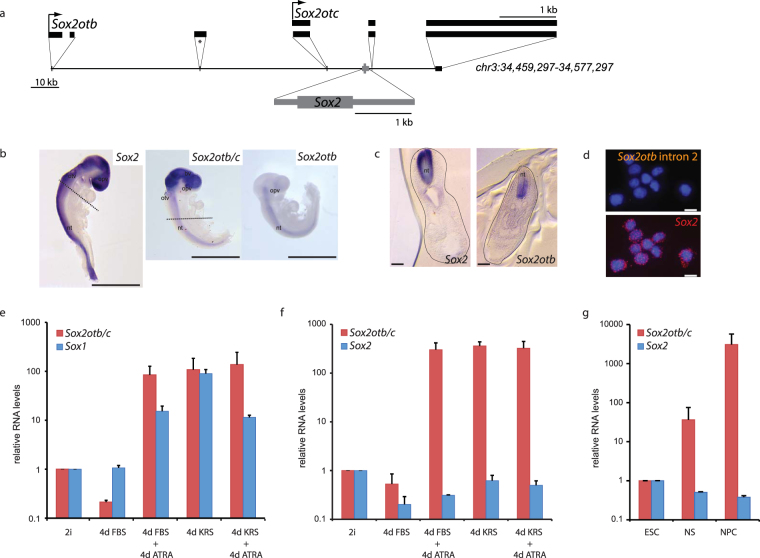
Co-expression of *Sox2otb/c* and *Sox2* during mouse neural development. (**a**) Schematic representation of the *Sox2* locus on mouse chromosome 3 (mm9 assembly). Depicted are the single exon gene *Sox2*, and the overlapping *Sox2otb* and *Soxtotc* genes. S*ox2otb* shares exons 4, 5 and 6 with *Sox2otc*. *Indicates  a newly identified exon. (**b**) Whole mount RNA *in situ* hybridization of E9.25 mouse embryos using antisense *Sox2, Sox2otbc and Sox2otb* RNA probes. Scale bar represents 1 mm. otv, otic vesicle; opv, optic vesicle; nt, neural tube; bv, brain vesicle. (**c**) Transverse sections of the embryos depicted in b. Dashed line in b indicates the level of the transverse section. nt, neural tube. Scale bar represents 100 μm. (**d**) smFISH on mouse ESCs using *Sox2otb* intron 2 (upper panel) or *Sox2* (lower panel) probe sets. Nuclei are stained with DAPI. (**e**) and (**f**) qRT-PCR analysis of *Sox2otb/c* and *Sox1* (**e**), or *Sox2otb/c* and *Sox2* (**f**) RNA levels during EB-mediated neural differentiation of mouse ESCs. Cells were cultured for 4 days in FBS or KSR containing medium followed by another 4 days in the same medium with 0.5 μM ATRA. (**g**) qRT-PCR analysis of *Sox2otb/c* and *Sox2* RNA levels in mouse ESCs, ESC-derived radial glia-like NS cells and NPCs derived from the lateral wall of the lateral ventricle of the adult mouse. Expression was first normalized against *β-Actin* (**e** and **f**) or *Myl6* (**g**), after which the relative expression to the expression in mouse ESCs was calculated. Values are mean + standard deviation (SD) of one representative out of 3 experiments and presented on a 10 log scale.

The *Sox2* gene is a single exon gene that is located in a gene desert on mouse chromsosome 3 (Fig. 1a). Apart from *Sox2* the only genes located within a 200 kb stretch of genomic DNA are presumably of non-coding nature. Two lncRNA genes (*Sox2otb* and *Sox2otc*) have been identified in this region^31^. The transcripts are initiated (~88 kb and ~11 kb) upstream of *Sox2* and are terminated ~40 kb downstream of *Sox2* (Fig. 1a). Transcriptome data, such as ESTs (expressed sequence tags) representing either *Sox2ot* transcript, have indicated that *Sox2ot* transcripts, like the flanking *Sox2* gene, are predominantly present in brain as well as cell lines of neural origin. The expression pattern points to a function of *Sox2ot* in neural development and neural physiology, possibly through a *Sox2*-related mechanism. We first validated the transcription *Sox2ot* genes in neural progenitor cells (NPCs) derived from the lateral wall of the lateral ventricle in adult mouse. Primers recognizing an exon of *Sox2otb* that also is the first exon of *Sox2otc* could amplify *Sox2ot* transcripts in early passage neurospheres (data not shown and Fig. 1g), which is in agreement with two recent studies^31,35^. Using 5′ RLM-RACE we confirmed the 5′ ends of *Sox2otb* and *Sox2otc* (Supplementary Fig. S1a). Full-length cDNA sequence analysis showed extensive splicing, which is arguably random as almost any possible exon conjunction was retrieved. The splicing is largely conserved in other mammals as was recently shown^34^. We identified one previously undescribed exon located between *Sox2otb* exon 2 and *Sox2otc* exon 1 (Fig. 1a). We analyzed the cDNA sequences for the presence of open reading frames (ORFs) through Coding Potential Calculator^36^, NCBI’s ORFfinder, and a translation initiation prediction program (ATGpr) but the outcome underscored the non-coding nature of all *Sox2otb* and *Sox2otc* splice variants (Supplementary Fig. S1d,e, and f). To test whether the transcripts can be translated into a polypeptide we performed in *vitro* transcription/translation assays using the largest, multi-exonic, *Sox2otb* and *Sox2otc* cDNA sequences, but we could not detect any Sox2ot polypeptides (Supplementary Fig. S1g). This result indicates that *Sox2otb* and *Sox2otc* are likely of non-coding nature as was suggested before^30,31^. However, our analyses do not fully exclude the generation of very small peptides with a function, which can be produced from presumed non-coding RNA transcripts^37^.

*Sox2ot* exonic and intronic sequences have been conserved between mammals and vertebrates (Supplementary Fig. S1c)^31^. The extent of conservation of genomic sequences between man and other vertebrates, like marsupials, is a measure of importance of these sequences for development. A larger evolutionary distance, i.e. between man and pufferfish (*Fugu rubripes)* diverging 450 million years ago, has been shown to be even more instrumental in uncovering coding as well as non-coding sequences crucial for proper development^38^. It was previously reported that the highest level of evolutionary conservation was observed in the promoter proximal regions of lncRNAs^39–41^. Likewise, the regions surrounding *Sox2otb* exon1 and *Sox2otc* exon 1, and not the exonic sequences, are highly conserved between man and *Fugu*. The high conservation of *Sox2ot* proximal promoter regions infers that *Sox2ot* sequences that govern transcription are more important during development than the transcript per se.

### Expression of *Sox2ot* during neural development

Since previous studies have indicated that *Sox2ot* expression positively correlates with *Sox2* RNA, we wished to test the correlative expression during neural development. We restricted the expression analysis to *Sox2otb, Sox2otc* and *Sox2* only. First we analyzed expression of *Sox2otb*, *Sox2otb* and *Sox2otc* (from here on referred to as *Sox2otb/c* because the riboprobe contains *Sox2otc* exon 1 sequence, which is also present *Sox2otb* transcripts), and *Sox2* in developing mouse embryos using RNA whole mount *in situ* hybridization (ISH). At 9.25 dpc *Sox2* expression is mainly restricted to the neural tube, developing brain, nasal placodes, otic vesicles and optic vesicles (Fig. 1b, Supplementary Fig. S2a,b) (sense controls in Supplementary Fig. S2c). In contrast, a probe recognizing *Sox2otb* showed an expression pattern limited to the ventral part of the neural tube and optic vesicle, whereas a probe hybridizing to *Sox2otb/c* showed additional expression in the developing brain and otic vesicles (Fig. 1b). The spatial and temporal specific expression patterns of *Sox2otb* and *Sox2otc* during neural development indicate that the independent *Sox2ot* transcripts may have different roles. Although it is difficult to robustly interpret co-localization data at the single cell level on basis of RNA ISH using independent single probe hybridizations, the ISH data show that *Sox2otb*, *Sox2otc* and *Sox2* are co-localized in tissues during neural development. To further investigate *Sox2otb*, *Sox2otc* and *Sox2* coexpression we analyzed *Sox2otb/c* and *Sox2* expression during the differentiation of mouse ESCs into neuroectoderm. In the tested feeder-independent and feeder-dependent wild type mouse ESC lines *Sox2otb/c* is very lowly expressed during maintenance. This is in sharp contrast with a previous study, which claimed abundant expression of *Sox2ot* in ESCs^31^. To further corroborate the low level of *Sox2ot* expression in ESCs we measured transcription of *Sox2ot* in mouse ESCs by single molecule FISH (smFISH) using a probe set lying in intron 2 of *Sox2otb*. smFISH has single molecule sensitivity^42^, yet, *Sox2otb* transcripts were very rare confirming the qRT-PCR results (Fig. 1d, positive control in Supplementary Fig. S2d). We observed a strong upregulation of *Sox2otb/c* upon neurectodermal differentiation using embryoid bodies (Fig. 1e, and Supplementary Fig. S2e). Upregulation coincides with the presence of neural progenitor/stem cells (NP/SCs) as measured through induction of *Sox1*, which is a very early and specific marker of the neuroectoderm lineage^43^. *Sox2ot* induction is all trans retinoic acid (ATRA) independent as neuroectodermal differentiation using knockout replacement serum (KRS) that is devoid of any form of retinol yielded a similar induction of *Sox2otb/c* (Fig. 1e). In more defined monolayer-based differentiation conditions *Sox2otb/c* was also induced upon neural differentiation (Supplementary Fig. S2d, and f), whereas BMP4-mediated differentiation towards mesendoderm failed to induce *Sox2otb/c* RNA levels (Supplementary Fig. S2g) indicating a primary role of *Sox2ot* in neural development. These results differ from the observations by Amaral *et al*., who have reported higher *Sox2ot* expression levels in mouse ESCs and enhanced *Sox2ot* transcription upon mesodermal commitment^31^. The discrepancies may be caused by differences in the used maintenance and differentiation protocols. Alternatively, a confounding factor may have been transcription initiation downstream of *Sox2* in certain cell types, which yields transcripts that encompass *Sox2ot* exon 6 sequences.

ESC-based neural differentiation cultures are a mixture of distinct cell types, which include ESCs, NSCs/NPCs, and early neurons. During neural differentiation *Sox2otb/c* RNA levels were rather negatively correlated with *Sox2* RNA levels (Fig. 1f) but the heterogeneic nature of the cultures thwarts to directly link *Sox2otb/c* levels to *Sox2* levels. To investigate whether *Sox2* levels are indeed negatively correlated with *Sox2otb/c* levels we measured the levels of *Sox2* and *Sox2otb/c* in *Sox2* heterozygous and homozygous ESC lines, in multiple monoclonal ESC-derived, radial glia-like neural stem (NS) cell lines generated from wild type mouse ESCs, and in neurosphere cultures of primary NPCs from the lateral ventricle of the adult mouse brain. NS cells express two to three-fold less *Sox2* RNA^44,45^ (Fig. 1g) but contain higher levels of *Sox2otb/c* RNA in comparison with mouse ESCs. Primary NPCs contain higher *Sox2otb/c* RNA levels, whereas *Sox2* levels are further reduced (Fig. 1g). In contrast to previous studies on *Sox2ot* expression in immortalized transformed cells^32–34^, we observed a negative correlation between *Sox2otb/c* and *Sox2* RNA levels (Spearman r = −0,7857, P-value = 0.048)(Supplementary Fig. S2h).

### Transcriptional activity of *Sox2ot* alters *Sox2* RNA levels in cis

**Figure 2:**
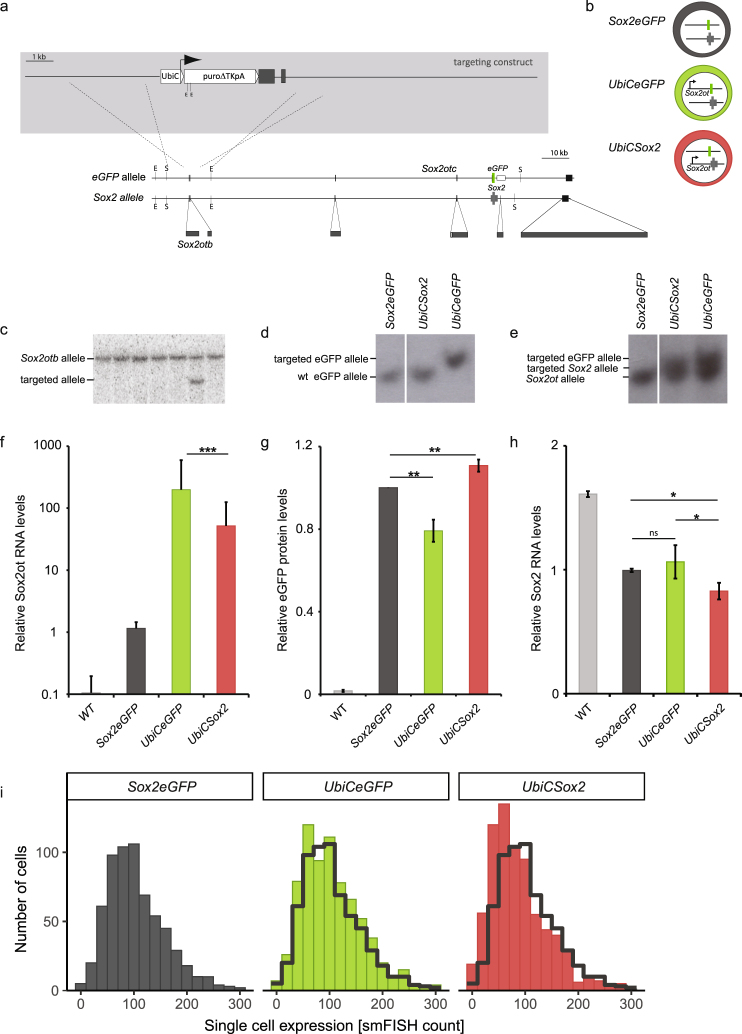
Allele-specific overexpression of endogenous *Sox2otb*. (**a**) Schematic view of the targeting strategy and targeting construct to generate allele-specific transcription of *Sox2ot*. R = EcoRV and S = SbfI restriction sites. (**b**) Illustration of the genetic possibilities after targeting the *Sox2eGFP* ESC line: *Sox2eGFP* (untargeted), *UbiCeGFP* (*Sox2ot* is expressed from the *eGFP* allele), or *UbiCSox2* (*Sox2ot* is expressed from the *Sox2* allele). (**c**) Southern blotting showing correctly recombined *Sox2eGFP* ESC clone using a 3′ probe (EcoRV restricted DNA). (**d**) and (**e**) Southern blot analysis showing correct targeting of the *eGFP* allele (*UbiCeGFP*) or *Sox2* (*UbiCSox2*) allele using eGFP (**d**) or *Sox2* (**e**) specific probes (SbfI restricted DNA). Full blots are shown in Supplementary Fig. S3a. (**f**) *Sox2otb* expression in *Sox2eGFP*, *UbiCeGFP*, and *UbiCSox2* cells as measured by qRT-PCR. (**g**) eGFP expression measured by flow cytometry in *Sox2eGFP*, *UbiCeGFP*, and *UbiCSox2* cells. (**h**) *Sox2* RNA levels in *Sox2eGFP*, *UbiCeGFP*, and *UbiCSox2* cells measured by qRT-PCR. (**i**) smFISH quantification of *Sox2* RNA copies per single cell in *Sox2eGFP*, *UbiCeGFP*, and *UbiCSox2* lines. The gray line depicts the distribution of *Sox2* in *Sox2eGFP* cells. ***P value < 0.002, **P value < 0.01 *P value < 0.05. Results are from three independent experiments using (sub)clones of *Sox2eGFP* (n = 2), *UbiCeGFP* (n = 3), and *UbiCSox2* (n = 2). Values are presented as mean +/− SD (g and h) or +SD (10 log scale (**f**)). qRT-PCR data were normalized against *β-Actin*, and relative levels to the levels in *Sox2eGFP* cells were determined. Statistical analysis was performed using the paired t-test, except for flow cytometry results (Wilcoxon signed-rank test) and smFISH results (Mann-Whitney U test).

Next we wondered whether the negative correlation between *Sox2ot* and *Sox2* is caused by a direct mechanism. Long non-coding RNAs are known to regulate neighboring genes in a variety of ways by either a cis (only the allele from which the lncRNA is transcribed is affected) or trans (the effect is independent of the allele from which the lncRNA is transcribed) mechanism. However, knocking out all three *Sox2ot* genes (*Sox2otb*, *Sox2otc*, and the 545 kb upstream of *Sox2* located *Sox2dot* (Supplementary Fig. S1b)) simultaneously is extremely difficult. Moreover, such a strategy would likely perturb ordinary locus regulation as removal of critical *Sox2ot* promoter sequences may delete important regulatory sequences that are key for correct expression of neighboring genes. To circumvent these pitfalls, we opted to enhance the transcriptional activity of *Sox2otb* in *Sox2* expressing cells that normally contain very low levels of *Sox2ot*. We introduced the human ubiquitin C (*UbiC*) promoter directly upstream of *Sox2otb* exon 1 by homologous recombination in mouse *Sox2eGFP* ESCs (Fig. 2a,c), which have one copy of *Sox2* replaced by eGFP^22^. Three clones contained an insertion of the *UbiC* promoter into the *eGFP* allele (*UbiCeGFP*) and two into the *Sox2* allele (*UbiCSox2*) (Fig. 2d, e). *Sox2otb* was highly transcribed in all targeted ESCs, albeit, levels were lower when the *UbiC* promoter was inserted into the *Sox2* allele, hinting towards the existence of an allele-specific modulatory mechanism (Fig. 2f).

If the negatively correlated expression of *Sox2* and *Sox2ot* is an immediate consequence of *Sox2ot* expression, an effect on *Sox2* as well as eGFP (trans regulation) or, on either *Sox2* or eGFP (cis regulation) should be evident in the targeted cells. Indeed, *Sox2ot* transcription resulted in a 20–30% reduction in *Sox2* or eGFP levels (Fig. 2g, h). However, reduced expression was solely observed for the gene (*Sox2* or eGFP) that was located on the targeted allele. These data demonstrate that *Sox2ot* transcription regulates *Sox2* transcription in cis. Although reductions were relatively moderate, a compensatory mechanism was activated in the ESCs that have decreased *Sox2* levels as illustrated by enhanced eGFP levels. This is reminiscent of the results in hybrid ESCs, in which allele-specific reduction of *Sox2* by deletion of the ESC prevalent transcriptional enhancer led to upregulation of *Sox2* from the unmodified allele^20^.

To determine whether the *Sox2* downregulation is specific for the whole population or whether only a proportion of the population contributed to the lower *Sox2* levels we quantified *Sox2* RNA at the single cell level by smFISH. smFISH allows us to count the expression of individual RNA molecules in individual cells, which reveals expression heterogeneity within the population. We measured *Sox2* levels in 700 cells of each ESC line (Fig. 2i, and Supplementary Fig. S3b). Only when *Sox2ot* was expressed from the *Sox2* allele we observed a ~20% reduction in the means (77 versus 96 (*Sox2eGFP*) or 98 (*UbiCeGFP*) transcripts). Moreover, the distribution of *Sox2* gene expression in *UbiCSox2* cells differed from *UbiCeGFP* and the parental *Sox2eGFP* cells (Mann-Whitney U; FDR = 3.19e-10 and FDR = 1.11e-10, respectively), whereas the distributions in *UbiCeGFP* and *Sox2eGFP* cells were comparable. This analysis confirmed that *Sox2* RNA levels are decreased when *Sox2ot* is transcribed from the same allele and showed that this effect is likely not restricted to a subpopulation of cells (Fig. 2i).

### Mouse ESCs overexpressing Sox2ot are very similar to wild type ESCs

**Figure 3:**
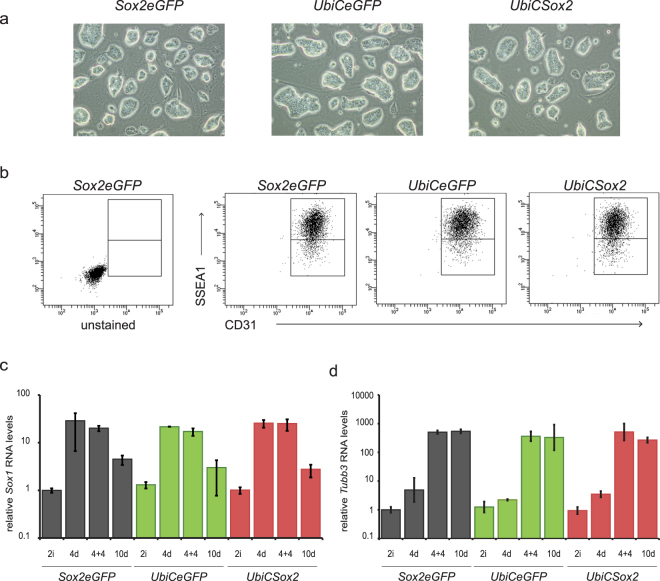
ESCs overexpressing endogenous *Sox2otb* are similar to *Sox2eGFP* ESCs. (**a**) Phase contrast pictures of *Sox2eGFP*, *UbiCeGFP*, and *UbiCSox2* cells cultured in 2i medium (100x magnification). (**b**) SSEA1 and CD31 expression in *Sox2eGFP*, *UbiCeGFP*, and *UbiCSox2* cells as measured by flow cytometry. (**c** and **d**) RNA levels of *Sox1* (**c**) and *Tubb3* (**d**) during EB-mediated neural differentiation of *Sox2eGFP*, *UbiCeGFP*, and *UbiCSox2* cells as measured by qRT-PCR. RNA levels were normalized against *β-actin*. RNA levels relative to the levels in *Sox2eGFP* cells are depicted on a 10 log scale. The results of one representative experiment (out of three independent experiments) using (sub)clones of *Sox2eGFP* (n = 2), *UbiceGFP* (n = 3), and *UbiCSox2* (n = 2) is depicted as mean +/− SD.

Next we investigated the effect of *Sox2ot* overexpression on the maintenance and differentiation of mouse ESCs. On basis of morphology we could not identify phenotypic differences between the parental *Sox2eGFP* ESCs and the *Sox2ot* overexpressing ESCs (Fig. 3a). The absence of a maintenance phenotype was underscored by the analysis of the expression of platelet endothelial cell activation marker CD31 (PECAM) and stage-specific embryonic antigen (SSEA1), which discriminates naïve and primed pluripotent cell states^44,45^. *Sox2eGFP* and *Sox2ot* overexpressing lines showed a similar and homogeneous CD31 expression profile, whereas SSEA1 was more heterogeneously expressed which is a normal feature of ESCs (Fig. 3b). Also the expression of other pluripotency genes like *Nanog* and *Oct4* was not altered (Supplementary Fig. S4a, S4b). In addition, prolonged passaging at a constant splitting ratio did not reveal gross differences in the expansion rate between *Sox2eGFP* and *Sox2otb* overexpressing ESCs (data not shown). Possibly this is due to adaptation of the *UbicSox2* ESCs to lower levels of *Sox2* RNA by acquiring more normal SOX2 protein levels (Supplementary Fig. S4c). Since *Sox2otb* is induced during the differentiation of ESCs into neuroectoderm we also investigated the effect of *Sox2otb* overexpression on neuroectodermal differentiation. Using EB-based differentiation protocols we could not detect quantitative or temporal differences in the generation of either NSCs or more mature Tubb3 positive cells (Fig. 3c,d). In addition, the differentiation into mesendoderm as determined by Brachyury expression is largely unaltered (Supplementary Fig. S4d). Taken together these results indicate that enhanced *Sox2ot* levels do not majorly alter the phenotype of ESCs and do not exert gross effects on the EB-based differentiation of mouse ESCs.

### *Sox2otb/c* is enriched in the nucleus but not associated to chromatin

**Figure 4:**
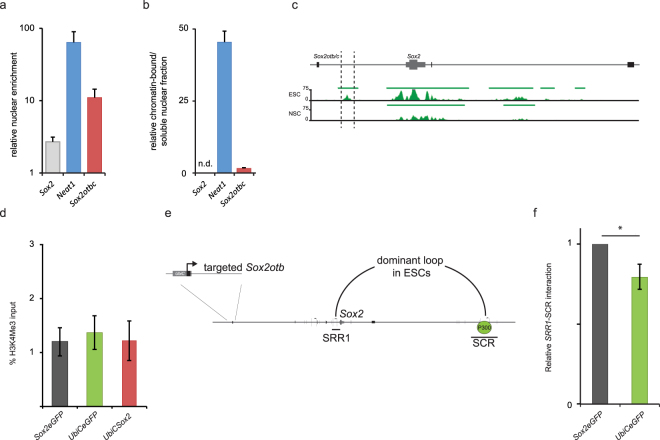
*Sox2* locus-specific H3K4 trimethylation and chromatin interactions in ESCs overexpressing *Sox2otb*. (**a**) Analysis of *Sox2ot* RNA localization in ESCs. *Sox2ot* is enriched in the nucleus when compared to *β-Actin* as determined by qRT-PCR after subcellular fractionation. The ratio (+SD) of nuclear/total RNA (200ng input) relative to that of *β-actin* is depicted on a 10 log scale. *Neat1* is a lncRNA that is enriched in the nucleus, and which is predominantly associated to chromatin^46^. (**b**) Analysis of the nuclear localization of *Sox2ot* in ESCs by qRT-PCR. The depicted ratio of chromatin bound RNA (+SD) is relative to that of *β-actin*. (**c**) Genome browser view of H3K4me3 density signals in the regulatory *Sox2* region of ESCs and ESC-derived NPCs^67^. For quantification of the difference see Supplementary Fig. 5Sa, b, and c. (**d**) H3K4me3 ChIP results for the region depicted between vertical lines in (**c**). Depicted H3K4me3 levels are relative to H3K4me3 levels of the housekeeping gene *Myl6*. (**e**) Schematic drawing of the dominant chromatin loop in ESCs formed by interaction of the *Sox2* proximal region (Sox2 regulatory region 1) (SRR1) with a P300 bound super enhancer (SCR) located ~110 kb downstream of *Sox2*. HindIII fragments and primers used are shown. (**f**) 3C chromatin conformation capture of the SRR1-SCR interaction depicted in (**e**). Values are relative to interactions of the *Sox2* intergenic region upstream of *Sox2otc*. Values are represented as mean +/− SD from three independent experiments (n = 10). *Paired t-test P value = 0.02.

Many lncRNAs that regulate transcription are enriched in the nucleus. We therefore investigated the cellular localization of *Sox2ot*. As our *Sox2ot* exonic smFISH probe set was not specific enough, we analyzed the cellular localization of *Sox2ot* RNA by cell fractionation and qRT-PCR. *Sox2ot* RNA was 4 times more enriched in the nucleus than *Sox2* RNA but 6 times less than *Neat1*, a lncRNA that is highly abundant in the nucleus^46^ (Fig. 4a). Next we examined whether *Sox2ot* is associated to the chromatin fraction. LncRNAs that function through a *trans*-acting mechanism are often found enriched in the chromatin fraction, like *Neat1* ^46^. In support of the observed in cis effect of *Sox2otb/c* we predominantly found *Sox2ot* RNA in the soluble nuclear fraction (Fig. 4b).

### H3K4 methylation is unaltered in *Sox2otb* overexpressing mouse ESCs

The allele-specific regulation of *Sox2* prompted us to investigate the nature of this regulation. A large group of cis-acting lncRNA transcripts represses genes by recruiting chromatin-modifying proteins that install a repressive histone mark such as H3K27me3 or H3K9me3, or by controlling H3K4 methylation^47^. To gain evidence for the existence of a *Sox2ot* dependent chromatin-modifying mechanism we compared H3K4me1, H3K4me2, H3K4me3, H3K9me3, and H3K27me3 chromatin marks in the region between the first exon of *Sox2otb* and the last exon of *Sox2otb/c* in cells expressing *Sox2* and *Sox2ot* at different ratios, i.e. ESCs and ESC-derived NPCs, using publicly available H3 methylation chromatin immunoprecipitation-sequencing (ChIP-seq) data sets (Fig. 4c, and Supplementary Fig. S5a and d). The only histone methylation profiles that are strongly altered between ESCs and ESC-derived NPCs are confined to a conserved region in the proximal enhancer/promoter region of *Sox2* ~4 kb downstream of the first exon of *Sox2otc* (Supplementary Fig. S5a,b, and c). In this region H3K27me3 and H3K4me3 were high in ESCs indicating a bivalent chromatin signature, which is linked to key developmental genes^48,49^. The bivalent histone status is lost in this region in ESC-derived NPCs. We wondered whether overexpression of *Sox2otb* would change the ESC chromatin into a more NPC-like chromatin regarding H3K4me3. We performed H3K4me3 ChIP assays for this region but did not observe differences in H3K4me3 between the cell lines (Fig. 4d). Although we did not rule out the involvement of other epigenomic changes, we decided to investigate other candidate regulatory mechanisms.

### *Sox2otb* transcription impairs the formation of the chromatin promoter-enhancer loop driving expression of *Sox2*

Development and homeostasis require coordinate regulation of neighboring genes through enhancers and locus control regions^50^. Chromatin looping enables transcription activation by juxtaposing locus control regions (LCRs), distal regulatory elements and promoter elements, and thus, function by bringing transcription factors, coactivators, and RNA polymerase II together. In ESCs multiple chromatin loops exist in the *Sox2* locus^51^. The most prevalent chromatin interaction is formed by the *Sox2* regulatory region 1 (SRR1) upstream of *Sox2* and a 13 kb super enhancer termed *Sox2* control region (SCR) located ~100 kb downstream of *Sox2* ^20,52^ (Fig. 4e). Deletion of this super enhancer decreases *Sox2* levels in mouse ESCs 6 to 9 fold^20,52^. Thus, if a decrease in *Sox2* levels were the consequence of *Sox2otb* mediated transcriptional interference the SRR1-SCR interaction would likely be diminished. Through chromosome conformation capture (3C) we analyzed whether the SRR1-SCR chromatin interaction was altered in *Sox2otb* overexpressing *(UbiCeGFP)* ESCs, which did not show altered *Sox2* levels, compared to parental *Sox2eGFP* ESCs. We indeed observed a lower frequency of SRR1-SCR interactions in *Sox2otb* overexpressing cells versus *Sox2eGFP* cells (Fig. 4f). In summary, transcriptional activity of *Sox2otb* negatively correlates with *Sox2* levels, and in addition, enhanced *Sox2otb* transcription correlates with reduced chromatin interactions between the upstream regulatory sequence of *Sox2* and the super enhancer of *Sox2* in mouse ESCs.

## Discussion

Through transcription analysis in combination with genetic modification of the endogenous *Sox2otb* locus we identified that transcriptional activity of *Sox2otb* represses *Sox2* expression in mouse ESCs. In contrast to our findings, previous studies in human cancer as well as cancer cell lines have demonstrated a positive correlation between *Sox2ot* and *Sox2* in certain but not all cell types investigated^32–34^. A quantitative and qualitative comparison of the published expression data is rather difficult due to the genomic positions of the primers used as the applied primer pairs recognize either a variety of *Sox2ot* splice variants or amplify only *Sox2ot* sequences downstream of *Sox2*. Nevertheless, the positive co-regulation of *Sox2* by *Sox2ot* has been strongly supported by ectopic overexpression or knockdown of *Sox2ot* pointing to a trans effect^32–34^. One may argue that transcription regulatory mechanisms of certain genes in human cells are different from those in murine cells, however, the strong conservation of the whole *Sox2ot* genomic region rather suggests a highly similar mode of operation. We believe that the disparities with the results obtained in this study are more likely caused by the differences in the cells analyzed, as gene regulation is very much cell type specific. In addition, cancer cells have undergone many epigenetic and genetic changes that interfere with the specificity and integrity of regular gene transcription programs^53^. Since we investigated early neural development using non-transformed mouse cells our data indicate that *Sox2* regulation during stem cell maintenance and differentiation is completely different from *Sox2* regulation in cancer cells.

Cis regulation of neighboring genes has been proposed to be an important function of many lncRNA genes, but up to now this has only been proven for a very small subset of lncRNAs predominantly involved in imprinting and X inactivation because of the more easily detectable allele-specific modifications^47^. In general, a major hurdle has been selection of an allele-specific genomic modification strategy to identify allele-specific differences that represent a *bona fide* phenotype. In addition, modification of lncRNA genes to study cis-acting mechanisms is rather challenging. Introducing single or small mutations that alter the function or expression of uncharacterized lncRNAs is very complicated due to the non-coding nature of these genes. Nevertheless, insertion of a strong polyadenylation signal that prematurely truncates the lncRNA transcript has been successfully exploited to gain insight into the requirement of the full-length lncRNA^27^. However, premature polyadenylation strategies do not allow analysis of the role of lncRNA transcription initiation or that of promoter/enhancer sequences. Instead deletion of presumed important regulatory regions may be considered to address their role. Recent genome editing advances using CRISPR/Cas9 have facilitated the deletion of genomic sequences^54^ but deletion of important promoter or exon sequences imposes the risk of removing important transcriptional regulatory regions of the neighboring genes, in particular, because lncRNAs are often transcribed from enhancer and promoter proximal sequences of adjacent genes. This may result in false attribution of the role of the modified lncRNA. As to *Sox2ot* the existence of at least three independent transcriptional initiation sites of *Sox2ot*, and possibly more as indicated by human CAGE datasets, would make the generation of a full knockout rather unrealistic. Moreover, one of the *Sox2ot* transcription initiation sites (that of *Sox2otc*) is located in regulatory sequences proximal of *Sox2*. Deletion of this genomic sequence may directly alter *Sox2* transcription independent of *Sox2otc*. As feasible alternative we created a promoter insertion that drives transcription of only one of the *Sox2ot* genes to study the role of *Sox2otb* overexpression in development and the regulation of *Sox2*. Using this overexpression system we demonstrate that the reduction in *Sox2* RNA levels is caused by allele-specific transcriptional activity of *Sox2otb*. The reduced levels of *Sox2* did not exert a loss of pluripotent stem cell self-renewal phenotype, as may have been expected, likely due to adaptation of the ESCs to decreased *Sox2* RNA levels. It is known that a decrease in *Sox2* levels in ESCs activates a feedback mechanism enhancing expression of *Sox2* ^20^. Also in the *UbicSox2* cells we observed upregulation of the other *Sox2* allele (here *eGFP* allele) indicating the activation of such feedback loop and the importance of having higher levels of Sox2. However, since the other allele is non-functional, enhanced expression of the other allele was ineffective. Instead the *UbicSox2* cells adapted to lower *Sox2* levels by regaining SOX2 to a level similar to that of the parental *Sox2eGFP* cells.

*Sox2* is also crucial for neuroectodermal differentiation of ESCs, and lower Sox2 levels favor mesendoderm commitment^9^. If the SOX2 protein levels would not have been enhanced upon adaptation a differentiation phenotype would have been expected in the cells that overexpress *Sox2otb* from the *Sox2* allele. Although Sox2 adaptation may have obscured an early neuroectodermal, Sox2-dependent differentiation defect, a Sox2-independent trans effect was not observed. Thus our results indicate that the main function of *Sox2otb* is cis regulation of *Sox2* rather than affecting cell physiology in trans via other routes. The importance of *Sox2ot* transcriptional activity is underscored by the genomic conservation of *Sox2ot* between mammals and *fugu*, which is much higher in *Sox2ot* promoter (proximal) sequences than exon sequences (Supplementary Fig. S1c).

**Figure 5:**
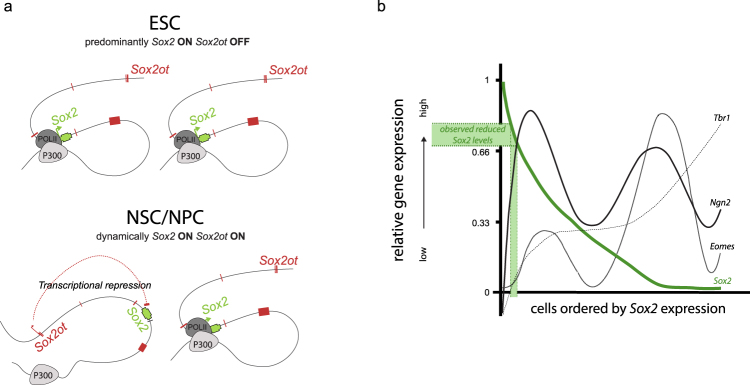
Proposed model of transcriptional interference to modulate *Sox2* levels during neural development. (**a**) Hypothetical model illustrating *Sox2* and *Sox2ot* transcription in ESCs and NSCs/NPCs. In ESCs *Sox2* (green) is predominantly transcribed, whereas *Sox2ot* (red) transcription is predominantly off. In NSCs *Sox2* and *Sox2ot* are transcribed in the same cell. On basis of our data we propose a dynamic on/off situation. If *Sox2ot* is transcribed *Sox2* transcription is repressed, and vice versa. (**b**) Adapted graph from a single cell RNA profiling study by Hagey and Muhr^55^ showing the influence of subtle reductions in *Sox2* on the expression of neuronal genes. The observed 20–30% reduction in *Sox2* transcription by *Sox2ot* transcription (indicated by green bars) lies at the threshold of the expression of neural genes *Tbr1* and *Eomes* and corresponds to a steep rise in the expression of the proneural gene *Ngn2* in cortical NSCs/NPCs.

The introduced *Sox2otb* transcriptional activity led to decreased *Sox2* transcription and reduced interaction of the *Sox2* proximal promoter region (SRR1) with the ESC-specific enhancer in this genomic region. However, we cannot rule out that other chromatin interactions are affected as well. In the presented heterozygous ESC model maximally 50% of a specific chromatin loop can be altered when considering an in cis effect. Therefore, only differences in very dominant chromatin loops, either the ones that are newly formed or the regular ones, are detectable. A hypothetical mechanism that would fit our observations is transcriptional repression by virtue of blocking recruitment of RNA polymerase II to the SSRI region (Fig. 5a). A very similar mechanism is exploited by *Airn*, which repress *Igf2r* by preventing RNA polymerase II recruitment to the *Igf2r* promoter^27^.

As well-balanced Sox2 protein levels are crucial for correct development of the distinct subsets of neurons in the neural tube^10,11^, it is tempting to speculate that during development the main function of *Sox2ot* is controlling *Sox2* levels. In this respect the 20–30% reduction in expression of *Sox2* RNA that we have observed may seem irrelevant. However, recently it became clear from single cell RNA sequence analysis in primary mouse cortical NSCs/NPCs that *Sox2* dosage regulates their division rate and controls their ability to maintain an undifferentiated state^55^. This study demonstrated that very modest decreases in *Sox2* levels in NSCs/NPCs are accompanied by rapid increments of the neuronal specification factor Neurogenin2 (Fig. 5b). Moreover, an approximately 20% reduced expression of *Sox2* (the same reduction as we observed in *Sox2ot* overexpressing ESCs) appeared to be a threshold for expression of the neural differentiation markers *Eomes* and *Tbr1* (Fig. 5b). These data indicate that a subtle decrease of *Sox2* may have a profound impact on the status of NSC/NPCs regarding their differentiation potential, and that *Sox2ot* transcription through the *Sox2* gene may render NSCs/NPCs more susceptible to neural differentiation.

We believe that the here proposed role of *Sox2ot* is likely conserved in numerous loci containing key differentiation genes. Transcriptome data have revealed that analogous overlapping transcripts are present in the *Sox1* and *Sox4* loci. It will be interesting to learn the underlying nature of these *Sox* regulatory mechanisms, to what extent this regulation exists in the mammalian genome, and how disruptions disturb development.

## Methods

### Cell culture

Mouse ES cell lines (E14-cl22^44^, E14 subclone IB10, R1, CCE, and *Sox2eGFP* ^22^ (parental mouse ESCs as well as the targeted clones) were cultured feeder-free or on irradiated mouse embryonic fibroblasts (MEFs) on 0.1% gelatinized tissue culture surface in DMEM containing 1 mM L-glutamine, 1x non-essential amino acids (NEAA), PenStrep 1%, 1000 U/ml human LIF (Peprotech), 50 μM *β*-mercaptoethanol and 15% mouse ESC tested fetal bovine serum (FBS) (Life Technologies). *Sox2eGFP* ESCs were kindly provided by the late Dr. L. Pevny, University of North Carolina, Chapel Hill. For ChIP, 3C, differentiation and qRT-PCR cells were first cultured for 4 passages in 2i medium^56^ (DMEM/F12/NeuralBasal, Glutamax, PenStrep 1%, human LIF 1000 U/ml (Peprotech), 50 μM *β*-mercaptoethanol, 0.5x B27 plus vitamin A (ThermoFisher), 0.5x N2 (ThermoFischer), 1 μM PD0325901 (Axon Medchem) and 3 μM CHIR99021 (Axon Medchem) and a FBS percentage that was gradually decreased from 15% to 1%. Cells were passaged using Trypsin/EDTA (0.05%/0.02%). Cells were maintained at 37 °C and 5% CO2. Prior to the experiments the quality of the cells was analyzed by flow cytometric analysis using anti-mouse SSEA1-BV421 (BD) and anti-mouse CD31-PerCPefluor710 (eBioscience) antibodies. SOX2 was measured by flow cytometry using a goat anti-Sox2 polyclonal antibody (SantaCruz, Biotechnology, sc-17319), in combination with an anti-goat-Alexa568 secondary antibody (Thermo Fisher Scientific). Staining was performed using  the fix & perm kit (Thermo Fisher Scientific) according the manufacturer’s instructions.

### Targeting Sox2eGFP mouse ES cells

Two independent homologous recombination experiments were performed using *Sox2OTb* targeting vectors containing UbiCloxPHyTKpAloxP or UbiClox2272PurDTKpAlox2272 selection cassettes. The selection modules were inserted 9 nucleotides upstream of the identified *Sox2OTb* transcription start site (chr 3: 34,459,297 NCBI37/mm9) into the genomic sequence (chr 3: 34,453,460–34,463,055 NCBI37/mm9) that was amplified from 129Ola genomic DNA using Phusion polymerase (NEB). The knockin constructs were introduced into *Sox2eGFP* ESCs by electroporation, and drug resistant clones were selected using hygromycin (110 μg/ml) or puromycin (1.5 μg/ml). Homologous recombinants were identified by Southern blot analysis of EcoRV restricted genomic DNA using ^32^P labelled *Sox2otb* flanking probes. In total, 465 colonies were screened for correct homologous recombination. Five correctly recombined clones were further investigated to identify whether the *Sox2* or *eGFP* allele was targeted. To this end SbfI restricted genomic DNA was separated by pulse field gel electroforesis (PFGE) and analyzed by Southern blotting using ^32^P labelled eGFP and *Sox2* probes. Three clones contained an insertion of the *UbiC* promoter into the *eGFP* allele (*UbiCeGFP*) and two into the *Sox2* allele (*UbiCSox2*).

### RNA *in situ* hybridization

Whole mount *in situ* hybridization was performed according standard protocols. In short, dissected E9.25 embryos (C57Bl/6) were fixed in 4% PFA O/N. Fixed embryos were twice washed in PBS 0.1% Tween-20 (Sigma) (PBST), and dehydrated by subsequent methanol washing steps (25-50-75 and 100% methanol). Dehydrated embryos were slowly rehydrated (10′ per step) at RT while rotating. After rehydration the brain vesicle was punctured and the surround membrane ruptured to prevent trapping of the riboprobes. Embryos were treated with proteinase K (10 mg/ml) for 10′, and gently rinsed in PBST. Next embryos were again fixed in 4% PFA and 0.2% glutaraldehyde for 20′ while rotating, washed in PBST, and incubated in 50% PBT/50% hybridization solution (HS) (HS: 50% formamide (Sigma), 1.3x SSC, pH 5.0 (Ambion), 5 mM EDTA, pH 8.0 (Ambion), 50 mg/ml yeast tRNA (Sigma), 0.2% Tween-20 (Sigma), 0.5% CHAPS (Thermo Fisher Scientific), and 100 mg/mL Heparin (Sigma)), and subsequently 100% HS. Riboprobes, generated by T7 polymerase *in vitro* transcription (antisense and sense *Sox2*, *Sox2OTb* and *Sox2OTb/c* digoxigenin labeled RNA probes (sequences in Table S1)), were added to HS and incubated for 20 hours at 70 °C. Embryos were washed 3 times with 2x SSC, 0.1% CHAPS, three times with 0.2x SSC, 0.1% CHAPS, and twice with 1x KTBT (50 mM TrisHCl, pH 7.5, 150 mM NaCl, 10 mM KCl and 1% Triton X-100). Embryos were incubated with 10 ug/ml of RNase A in KTBT for 30 min. at 37 °C, blocked with 2% blocking solution (Roche), and 20% heat inactivated sheep serum, and subsequently O/N incubated with AP conjugated a-DIG, Fab fragment (Sigma) in the same blocking buffer at 4 °C. Embryos were 5 times washed in 0.1% Tween-20 and 1 mM levisamole (Roche) in ddH2O, and subsequently stained in 1x BM purple (Roche) plus 0.1% Tween, 1 mM levamisole. Reaction was stopped by washing in ddH2O.

Whole mount stained embryos, were embedded in 2% agarose and cross-sectioned on a vibratome (Leica). Mice were maintained under specific-pathogen-free conditions. All animal experiments were approved by the Animal Experiments Committee of the LUMC performed to the recommendations and guidelines set by the LUMC and by the Dutch Experiments on Animals Act that serves the implementation of guidelines on the protection of experimental animals by the Council of Europe.

### RNA-linker mediated (RLM)-RACE and *in vitro* transcription translation

The used RLM-RACE procedure has been extensively described elsewhere^57^. *Sox2ot* reverse primers were located in exon 1 of *Sox2otc*. *In vitro* transcription/translation of human *TP53* and the full-length *Sox2otb* and *Sox2otc* cDNA sequences was performed using TNT® Quick Coupled Transcription/Translation System (Promega) according the manufacturer’s protocol. ^35^M labeled proteins were separated on 5–15% and 20% polyacrylamide gels.

### ESC differentiation

For embryoid body (EB) differentiation the original protocol was slightly adapted^58^. For neural differentiation: ESCs were seeded as a single cell suspension at a concentration of 100,000-200,000 cells/ml in ESC media containing FBS (as in the original protocol) or knockout serum replacement (KSR)^59^ lacking hLIF and 2i on ultra-low attachment plates (Corning). After 4 days of culture all trans retinoic acid (ATRA) (Sigma) or the synthetic substitute EC23 (Abcam) was added to the media at a concentration of 0.5 μM. Media was changed once every two days. For mesendodermal differentiation, aggregated ESCs were cultured in 2i media containing 3 μM CHIR99021 but without PD0325901, hLIF, and FBS as has been described for monolayer differentiation^9^. 3 ½ days after addition of CHIR99021 EBs were manually dissociated using the embryoid body dissociation kit (Miltenyi Biotech) according the manufacturer’s instructions. Cells were stained for Oct4 and Brachyury using mouse anti-Oct4-BV421 (BD) and goat anti-Brachyury (SC-17745, SantaCruz) and a secondary donkey anti goat Alexa568 antibody (Thermo Fisher Scientific) using the fix & perm kit (Thermo Fisher Scientific) according the manufacturer's protocol. Oct4 and Brachyury expression was measured on a LSRII flow cytometer (BD).

For monolayer differentiation we adapted the protocol used by Engberg *et al*.^60^. In brief, mouse ESCs were seeded at a density of 15,000 cells/cm^2^ onto 0.1% gelatin (Sigma) coated dishes in 2i media, lacking hLIF and 2i, but containing 1% FBS. Media was replaced with DMEM/F12/Neuralbasal containing L-glutamine (ThermoFisher), PenStrep 1%, 1x N2 (ThermoFisher), and 1x B27 without vitamin A (ThermoFisher), and ATRA (Sigma) or EC23 (Abcam), or hBMP4 (Peprotech) at the concentrations indicated, 12 hours after seeding the cells. Cells were cultured for the indicated periods and media was replaced every two days. NS cell lines were generated from different ESC lines using N2B27 media as described elsewhere^61^. One of the clones has been extensively characterized^44^.

### RNA isolation and quantitative PCR analysis

Total RNA was isolated directly from the cells using Trizol (Life technologies) or NucleoSpin® columns (Macherey-Nagel). Following DNaseI treatment (Roche), cDNA was generated from 100–500 ng RNA using Transcriptor reverse transcriptase (Roche) and random hexamers or an oligod(T) primer according the manufacturer’s protocol. After the samples had been checked for genomic DNA contaminations, cDNA was measured quantitatively on a Bio-Rad CFX96 using SensiFAST^TM^ Sybr green PCR mix (Bioline) and the primers listed in Supplementary Table S1. All primers were tested for a comparable and linear amplification efficiency using a dilution series of cDNA or gDNA. RNA levels were normalized against *β-actin* and 18 *S*, which yielded similar outcomes. For direct quantitative comparison of expression levels between ESCs and NS cells levels were normalized against housekeeping gene *Myl6* because *Myl6* expression is unaltered between ESCs and NSCs^44^. All measurements were performed in triplicate. Relative expression was calculated using the comparative Ct method, known as the 2–[delta][delta]Ct method, where [delta][delta]Ct = [delta]Ct(sample) - [delta]Ct(reference). Dependent on the experiment, the reference samples were the 2i samples (also described as day 0 of differentiation), or the parental ESC line *Sox2eGFP*.

### Single molecule fluorescence *in situ* hybridization (smFISH)

Mouse ESCs were cultured in 2i medium or differentiated in N2B27 media without additives for 4 days as described above. Cells were detached with Accutase (Gibco), resuspended in serum containing medium, and fixed by adding paraformaldehyde to an end-concentration of 4% and subsequent incubation for 12 minutes at RT. Fixed cells were pelleted by a 3′ centrifugation and subsequently resuspended in 70% ethanol. Samples were stored at 4 °C until use. smFISH of *Sox2* (Stellaris VSMF-3075-5-BS probe set) was performed exactly as before^62^ and signals were quantified using custom MATLAB scripts. *Sox2ot* transcription was determined using a custom probe set covering *Sox2otb* intron 2, which was designed by homemade MATLAB scripts.

### Chromatin immunoprecipitation (ChIP) and 3C conformation capture

The chromatin of a single cell suspension of mouse ESCs was crosslinked in ESC medium containing 1% formaldehyde. Protocols used were previously described by Lee *et al*.^63^ (ChIP) and Stadhouders *et al*.^64^ (3C). For ChIP: the nuclear fraction was sonicated for 9 minutes (30″ on, 30″ off) using a Biorupter UCD-200 (Diagnode). After sonication, H3K4me3 chromatin was precipitated overnight at 4 °C in 0.1% fraction V BSA, protease inhibitors (Roche), 16.7 mM trisHCl, 167 mM NaCl, 1.25 mM EDTA, 0.01% SDS, 1% Triton X-100, Dynabeads Protein G (ThermoFisher) and 1 ug H3K4me3 rabbit polyclonal antibody (Diagenode). Chromatin was eluted in 1% SDS, and 0.1 M NaHCO3, de-crosslinked at 65 °C for 8–12 hours, treated with RNAse A and ProtK, and purified using phenol/chloroform extraction. Mouse insulin promoter primers and *Myl6* primers were used as negative control and positive/normalization control, respectively. For 3C: chromatin was restricted with HindIII (Fermentas) for 24 hours and O/N ligated at 16 °C. Chromatin was de-crosslinked at 65 °C for 8–12 hours, treated with RNAse A and ProtK, and purified using phenol/chloroform extraction. Quality and quantity of DNA was checked by a linear amplification of Sox2UTR genomic sequences. Ligation efficiencies were checked through amplification of ESC-specific *Dppa2* chromatin loop^65^.

### Subcellular fractionation

Cell fractionation: mouse ESCs were divided into two fractions and used for either total RNA isolation or nuclear RNA isolation. Nuclear RNA was isolated as previously described^57^. In brief, cells were lysed and nuclei were pelleted after centrifugation (1350 g at 4 °C for 5 min). Cells (total RNA) or nuclei (nuclear RNA) were lysed using RA1 RNA lysis buffer (Macherey-Nagel) and RNA was isolated on NucleoSpin® columns (Macherey-Nagel) according to the manufacturer’s instructions. 200 ng of RNA was used in the reverse transcription reaction that was performed as described above.

Nuclear fractionation: Fractionation of the nucleus was performed as described by Werner *et al*.^66^ In brief, crude nuclei were resuspended in 250 μl NRB (20 mM HEPES pH 7.5, 50% Glycerol, 75 mM NaCl, 1 mM DTT, 1x protease inhibitor cocktail) and centrifuged for 5′ at 500 g at 4 °C. The pellet was again resuspended in 250 μl NRB and 1 volume of NUN buffer ((20 mM HEPES, 300 mM NaCl, 1 M Urea, 1% NP-40 Substitute, 10 mM MgCl2, 1 mM DTT) was added, followed by a 5′incubation on ice after which the suspension was centrifuged (1,200 g, 5 min, 4 °C). The soluble fraction supernatant was transferred to a tube and the pellet was resuspended in 1 ml buffer A (20 mM HEPES pH 7.5, 10 mM KCl, 10% glycerol, 340 mM sucrose, 4 mM MgCl2, 1 mM DTT, and 1x Protease Inhibitor Cocktail (Roche) and centrifuged (1,200 g, 5 min, 4 °C). The chromatin pellet was resuspended in 50 μl buffer A, and 500 μl Trizol (Life technologies) was added. Trizol was added as well to the soluble nuclear fraction. Subsequently, RNA was extracted following the manufacturer’s guidelines. cDNA was generated as described above.

## Electronic supplementary material

Supplementary Information
