# Supplementary material for: Allele-specific repression of Sox2 through the long non-coding RNA Sox2ot
Source: Sci Rep. 2018 Jan 10;8:386. doi: 10.1038/s41598-017-18649-4 (PMC5762901; doi:10.1038/s41598-017-18649-4)
Supplement: Supplementary file 1 — Supplementary Information [file 41598_2017_18649_MOESM1_ESM.pdf]

Allele-specific repression of *Sox2* through the long non-coding RNA *Sox2ot*

Tobias C. Messemaker[1,2], Selina M. van Leeuwen[1], Patrick van den Berg[3], Anke E.J. ‘t Jong[1], Robert-Jan Palstra[4], Rob C. Hoeben[1], Stefan Semrau[3] and Harald M.M. Mikkers[1]

Supplementary Figure 1S plus legends

Supplementary Figure 2S plus legends

Supplementary Figure 3S plus legends

Supplementary Figure 4S plus legends

Supplementary Figure 5S plus legends

Supplementary Table 1S

Supplementary methods info

Supplementary Figure S1

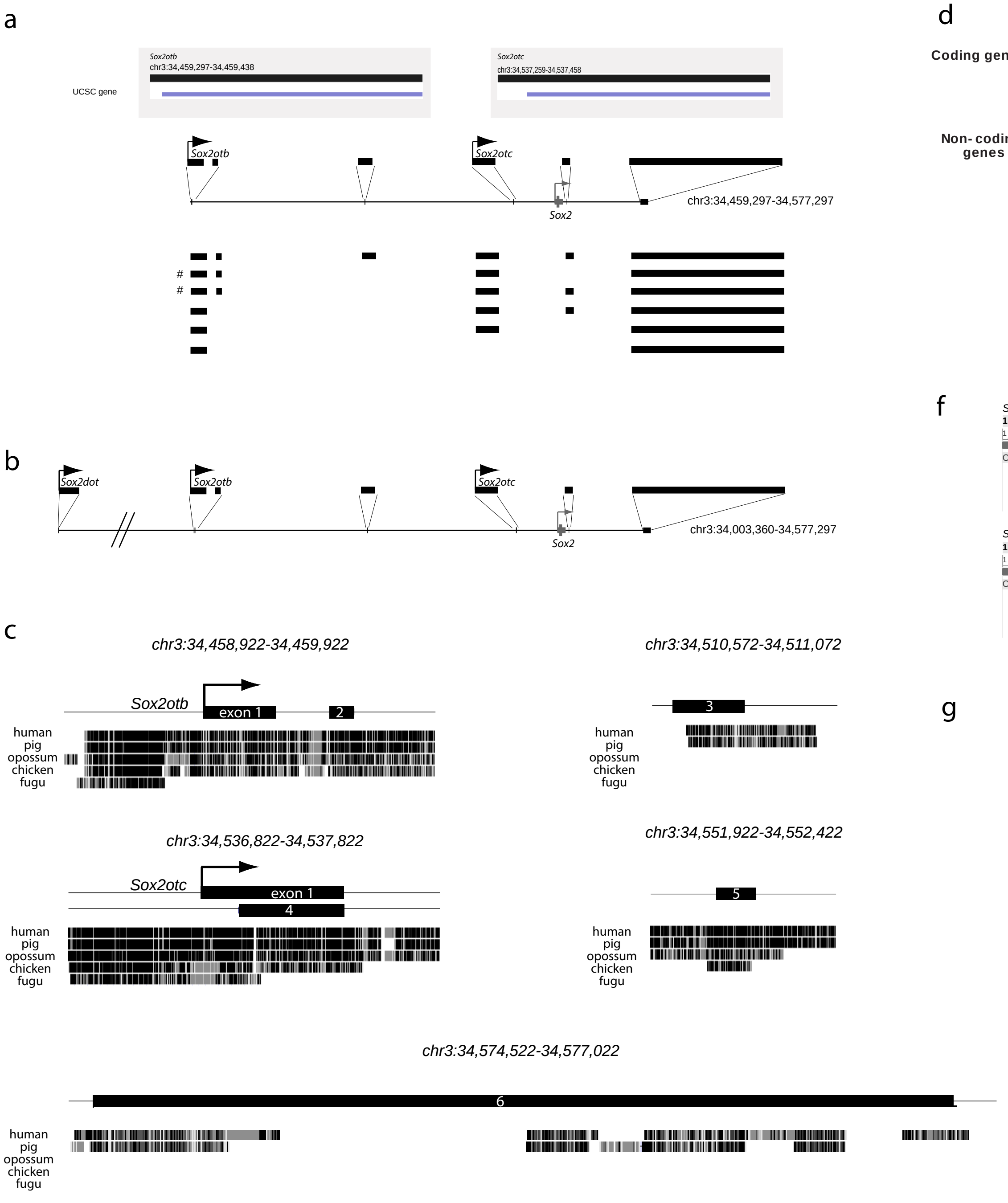

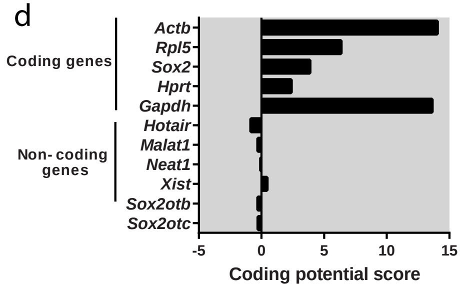

e

Sox2otb

No. of ATG from 5'end	Reliability	Frame	Kozak sequence	Start (bp)	End (bp)	ORF length (aa)	Sequence
6	0.23	1	tXXATGG	382	699	106	MEKEGGSASSFNSFCPVRSDPAWADERLRSLHVQCFL PANQTATRQHPDLAWSPRVQACSPKSENLCSWYTQKR SQATDILQKKALNSEIEPRNCTFLKIRLRESG
13	0.13	2	AXXATGt	929	1096	56	MYLITQQGQLELYPSVAILNKRRDRQTDRQTDRQAGRQ ADRGRDRQTETEKQRNDS
23	0.09	3	tXXATGt	1824	1964	47	MCILSQRACGDYKTVKIGHSRQHLRDQLRTLTCHFCVTF GVAVIIFL
1	0.08	2	cXXATGG	122	238	39	MGKVPGLVCWDRPHPQELWAVPRSKPDRFLQNSGHP WDE
15	0.06	1	tXXATGc	1150	1221	24	MPVVSALEQIKQESLKTKVSLDHW

Sox2otc

No. of ATG from 5'end	Reliability	Frame	Kozak sequence	Start (bp)	End (bp)	ORF length (aa)	Sequence
3	0.23	1	tXXATGG	232	549	106	MEKEGGSASSFNSFCPVRSDPAWADERLRSLHVQCFL PANQTATRQHPDLAWSPRVQACSPKSENLCSWYTQKR SQATDILQKKALNSEIEPRNCTFLKIRLRESG
10	0.13	2	AXXATGt	779	946	56	MYLITQQGQLELYPSVAILNKRRDRQTDRQTDRQAGRQ ADRGRDRQTETEKQRNDS
20	0.09	3	tXXATGt	1674	1814	47	MCILSQRACGDYKTVKIGHSRQHLRDQLRTLTCHFCVTF GVAVIIFL
12	0.06	1	tXXATGc	1000	1071	24	MPVVSALEQIKQESLKTKVSLDHW
11	0.06	3	GXXATGa	939	986	16	MIPELFLKINFKIQIN

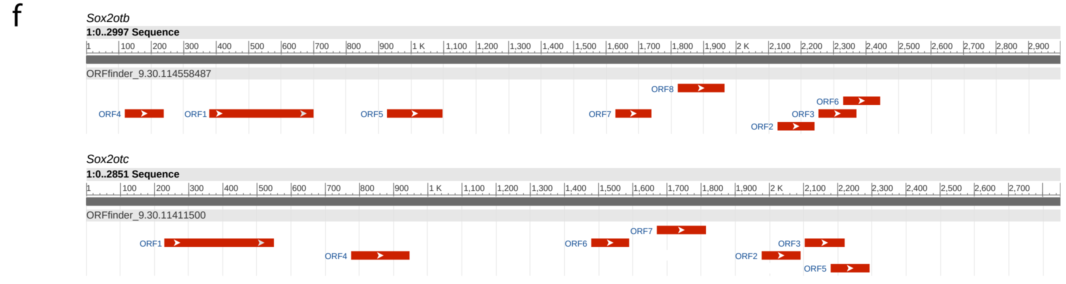

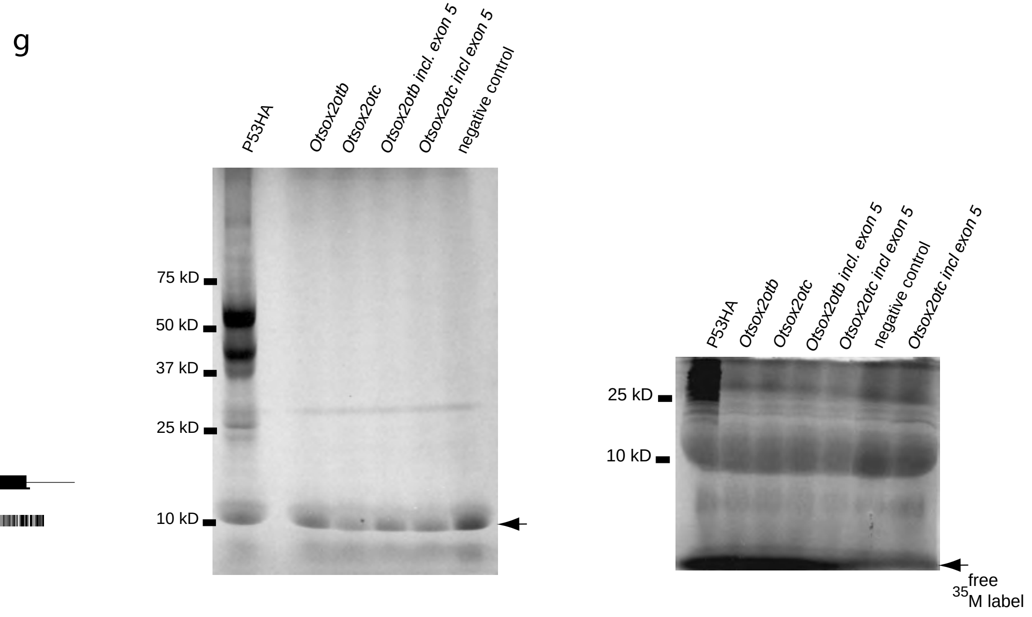

Supplementary Fig. S1.

Characterization of *Soxot* transcripts. (a) Genome browser view of *Sox2otb* and*Sox2otc.*Sequences that we isolated from primary mouse neurospheres were aligned to the mouse genome (Mm9). Multiple *Sox2ot* splice variants were identified. In addition, RLM-RACE identified two transcription start sites (TSSs) that are further upstream than previously identified TSSs. (b) Genome browser view of *Sox2dot*, for which we could not isolate 5' capped RNA sequences*.* (c) Multiple sequence alignments showing the conservation of the genomic sequence surrounding the exons of *Sox2otb* and *Sox2otc* from human to *fugu*. *Sox2ot* promoter sequences have been more strongly conserved. The grayscale of the blocks indicates the conservation strength. d) Sequence analysis of all splice variants did not show any coding potential using the coding potential calculator (CPC). (e) Predicted ATG protein starts for *Sox2otb* (top) and *Sox2otc* (bottom) using a translation initiation prediction program (http://atgpr.dbcls.jp/). Specificity and sensitivity of this program crossed at 46% at a ATGpr score threshold of 0.33 indicating that translation initiation of *Sox2ot* transcripts is rather unlikely. (f) Possible ORFs in *Sox2otb* and *Sox2otc* using NCBI's ORFfinder. (g) In *vitro* translation of the transcripts (marked # in (a)) did not reveal any polypeptide generation on a 8-20% SDS polyacrylamide gradient gel (left) or a 20% SDS polyacrylamide gel (right).

Supplementary Figure S2

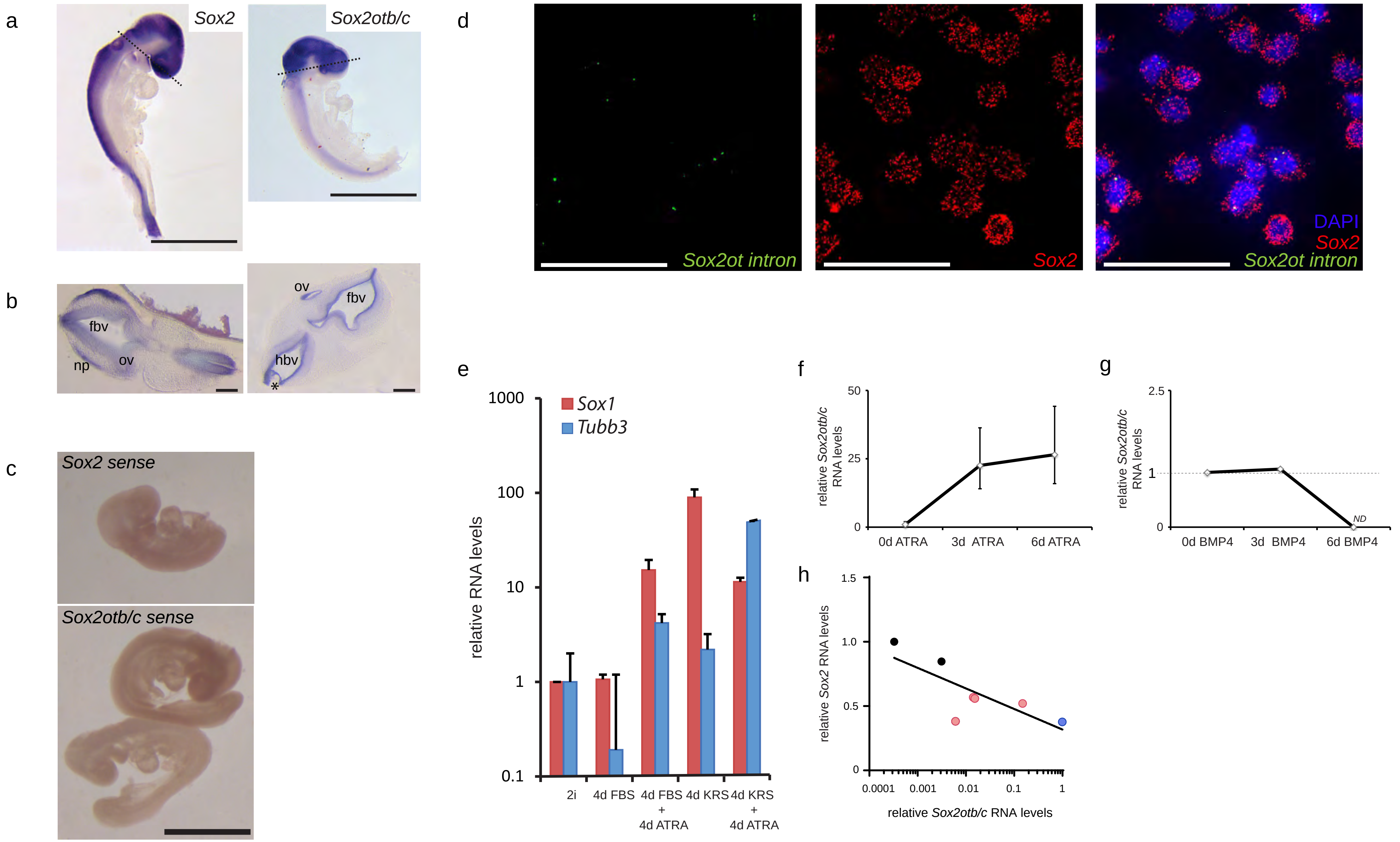

Supplementary Fig. S2.

***Sox2ot* RNA expression is correlated with *Sox2* RNA expression.** (a) Wholemount RNA ISH of E9.25 mouse embryos using antisense *Sox2* and *Soxot b/c* RNA probes. Scale bar represents 1 mm. Dotted line indicates the position and plane of the transverse sections depicted in b. Scale bar represents 100 μm. (b) transverse sections of whole mounts depicted in a. op, olfactory placode; opv, optic vesicle; bv, brain vesicle; fbv, forebrain vesicle; hbv, hindbrain vesicle; * indicates the location where the brain vesicle was punctured to eliminate probe trapping. (c) Whole mount RNA ISH of E9.25 mouse embryos using *Sox2* and *Soxot b/c* sense control RNA probes. Scale bar represents 1 mm. (d) smFISH with *Sox2ot* intron 2 probe on day 4of N2B27 guided monolayer differentiation of wildtype mouse ESCs into the neural lineage. *Sox2ot* signal in green. *Sox2* signal in red. Nuclei were visualized using DAPI. Scale bar represents 50 μM. (e) Induction of *Sox1* is accompanied by upregulation of the early neuronal marker *Tubb3* as demonstrated by qRT-PCR analysis. Averages are from one representative differentiation experiment using two independent mouse ESC lines. Cells were differentiated as embryoid bodies in the presence of fetal bovine serum (FBS) or knockout replacement serum (KRS) and after 4 days ATRA was added. SDs are shown. Raw qRT-PCR values were normalized against *β-Actin*, and results are represented as fold induction to the levels of *Sox1* and *Tubb3* prior to differentiation (d0 in 2i medium). (f and g) Monolayer differentiation of mouse ESCs. (f) *Sox2otbc* expression is induced during neural differentiation in N2B27 + ATRA medium. (g) However not during BMP4-mediated mesendoderm differentiation of mESCs. Averages + SD are from one representative differentiation experiment using two independent mouse ESC lines. Raw qRT-PCR values were normalized against *β-Actin*, and results are represented as fold induction to the levels of *Sox2otb/c* prior to differentiation (d0). (h) non-linear regression plot of relative *Sox2* and *Sox2ot* levels showing a negative correlation. Black circles (mouse ESC lines), red circles (mouse NS lines), light red circle (NPC enriched differentiation), and blue circle (primary NPCs).

Supplementary Figure S3

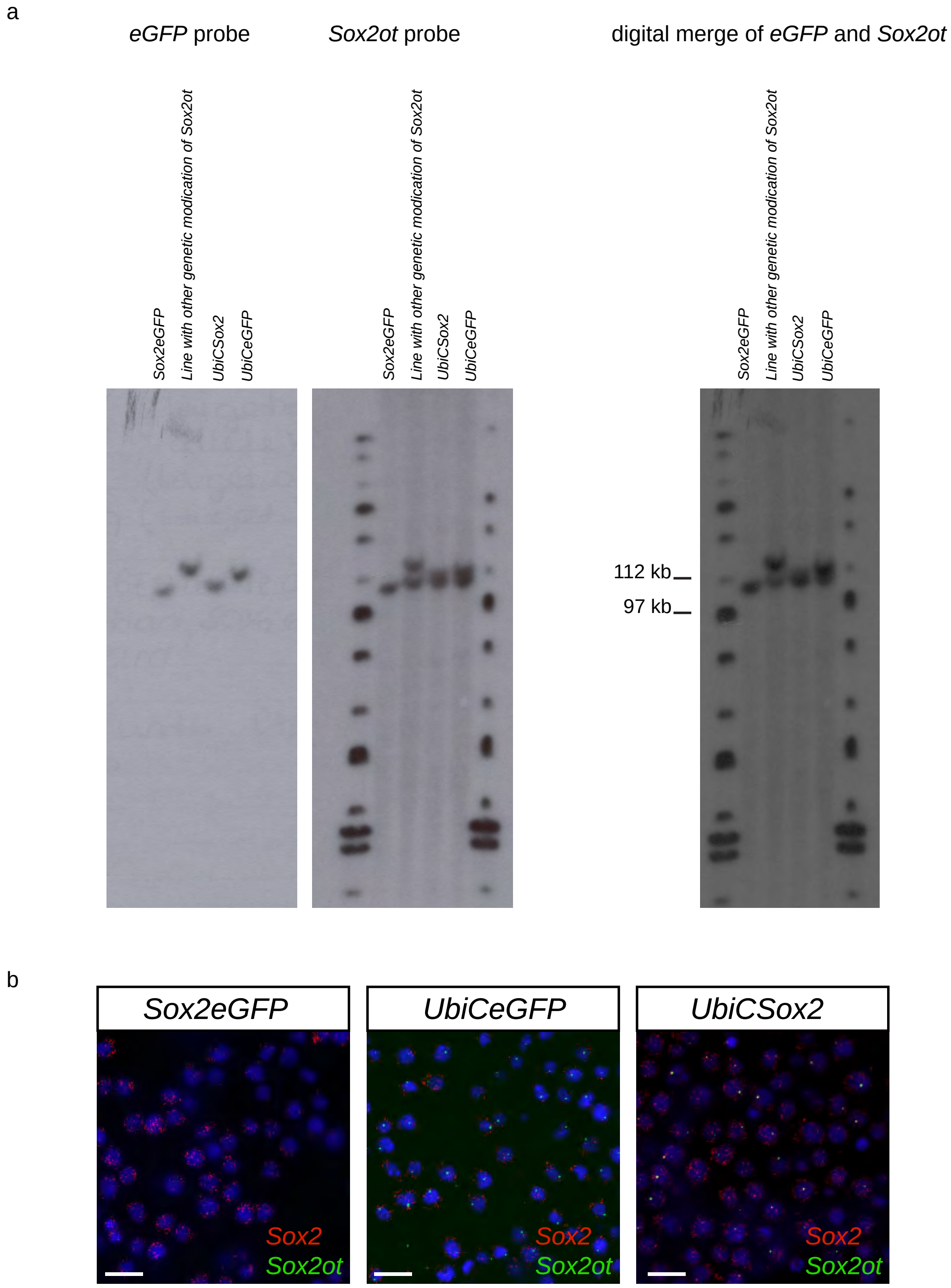

Analysis of *Sox2ot* knockin *Sox2eGFP* ESCs. Original PFGE Southern blots belonging to Fig. 2d and 2e showing allele specific targeting of *Sox2eGFP* ESCs (a). Left panel *eGFP* probe, middle panel *Sox2ot* probe, right panel overlay of both blots. (b) *Sox2* (red) and *Sox2ot* (green) smFISH results in *Sox2eGFP* (left), *UbiCeGFP* (middle) *and UbiCSox2* ESCs (right). Nuclei are visualized by DAPI. Scale bar represents 20 μM.

Supplementary Figure S4

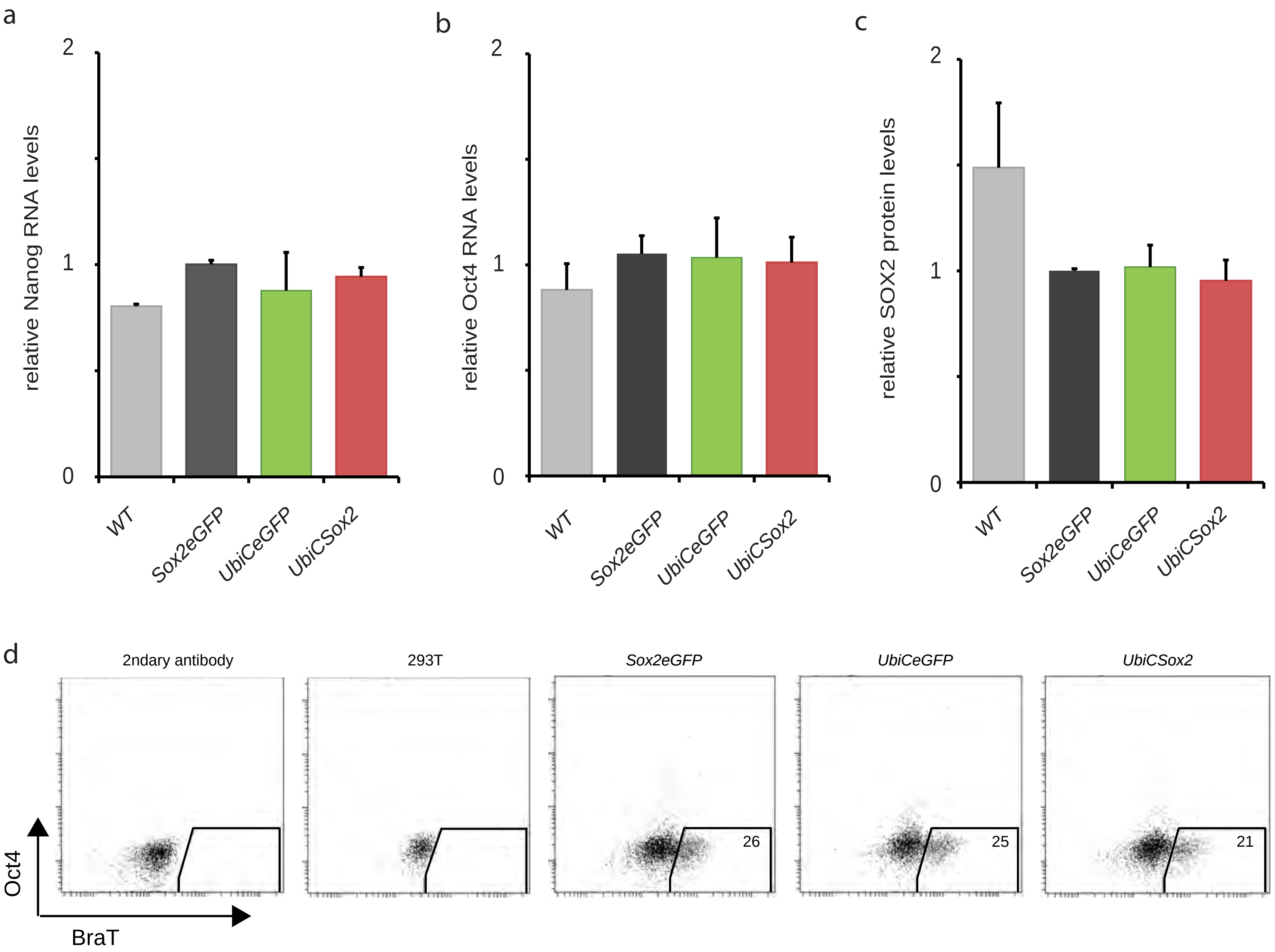

***Sox2otb* overexpressing mouse ESCs are very similar to the parental *Sox2eGFP* mouse ESCs.** Comparing relative RNA levels of *Nanog* (a) or *Oct4* (b) in WT, Sox2eGFP, *UbiCeGFP* and *UbiCSox2* ESCs. Values (+SD) are from three independent experiments of two mESC lines per genotype. (c) The mean SOX2 protein levels in WT, Sox2eGFP, *UbiCeGFP* and *UbiCSox2* ESCs as measured by flow cytometry. (d) Flow cytometric analysis of Brachyury positive cells upon CHIR99021-mediated mesendodermal differentiation of *Sox2eGFP*, *UbiCeGFP* and *UbiCSox2* cell lines at day 3.5 of differentiation. Controls are secondary antibody only and 293T cells which are negative for Brachyury.

Supplementary Figure S5

a

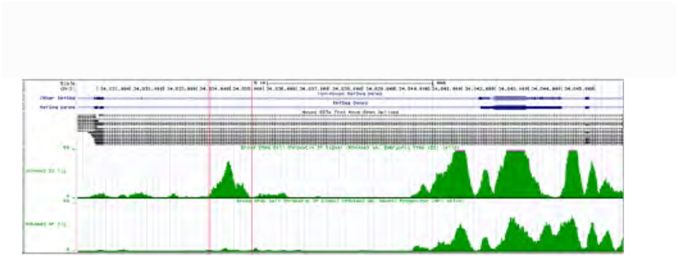

b

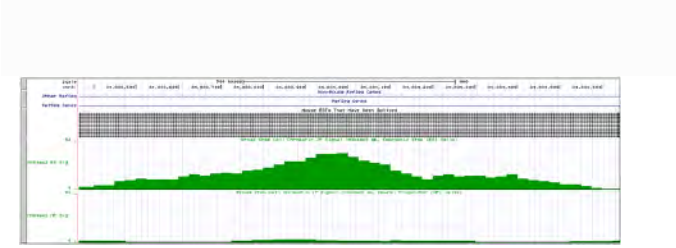

Database: mm8						Table: broadStemChipSignalH3K4Es					
Chrom	Data start	Data end	# of Data values	Each data value spans # bases	Bases covered	Minimum	Maximum	Range	Mean	Variance	Standard deviation
chr3	34833376	34834625	50	25	1,250 (97.96%)	0	38.2323	38.2323	16.332	96.1649	9.80637

Database: mm8						Table: broadStemChipSignalH3K4Np					
Chrom	Data start	Data end	# of Data values	Each data value spans # bases	Bases covered	Minimum	Maximum	Range	Mean	Variance	Standard deviation
chr3	34833376	34834625	50	25	1,250 (97.96%)	0	3.16142	3.16142	1.24157	1.09045	1.04425

c

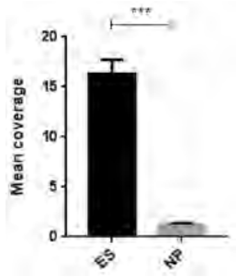

d

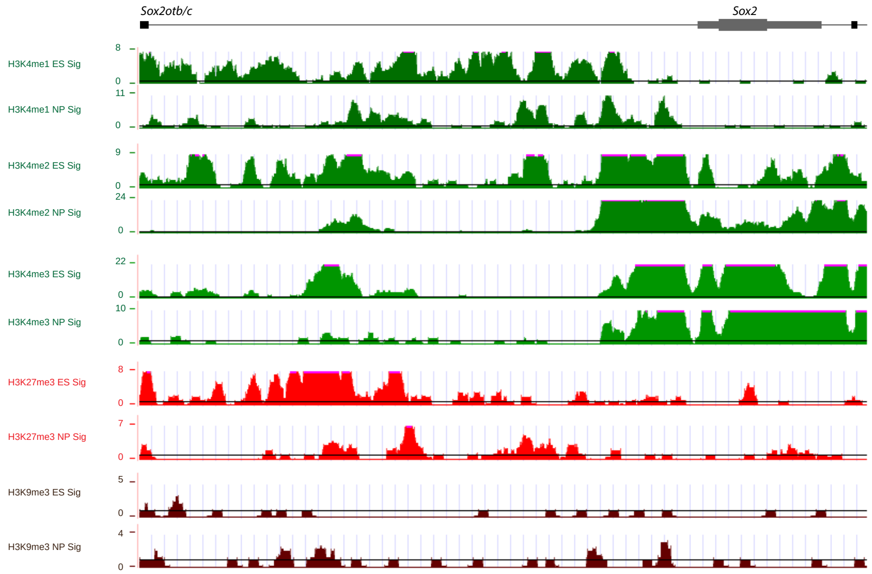

Assessing the differences in histone modifcations between ESCs and NPCs. (a) First step: visual assessment of the differences, here depicted as density signal, of H3K4me3 ChIP-seq reads aligning to the region mouse chr 3: chr3:34,829,397-34,845,851 (mm8). (b) The mean H3K4me3 coverage and standard deviation of 25-basepair regions were extracted from UCSC over a 1250 bp region resulting in 50 data values. (c) The mean H3K4me3 coverage ± SEM for this 1250 bp region is depicted for embryonic stem cells (ES) and neural progenitor cells (NP). Students T-test was used to determine the differences. (d) Genome browser view of mouse chr3: 34,830,000-34,845,000 (mm8) depicting H3K4me1, H3K4me2, H3K4me3, H3K9me3 and H3K27me3 density peaks in chromatin of mouse ESCs and ESC-derived NPCs (Broad ChIPseq data).

Messemaker_Supplementary Table S1

primer name	Sequence
**Probes for DNA and RNA hybridisations**	
eGFP2.1for	5’-GAGCTGGACGGCGACGTAAACG-3’
eGFP2.1rev	5’-CGCTTCTCGTTGGGGTCTTTGCT-3’
mSox2otbforQ	5’-TTGATCCTCTGATGGGGAAG-3’
mSox2otbrev	5’-TTACACCAGCCTCCAAGACC-3’
mSox2OTbcforQ	5’-CTCGTCAGCCCAAGCTGGATC-3’
mSox2OTbcrevQ	5’-CTCGTCAGCCCAAGCTGGATC-3’
mSox2for	5’-GTTCTAGTGGTACGTTAGGCCTTC-3’
mSox2rev	5’-GGACATTTGATTGCCATGTTTATCTCG-3’
Sox2otTargetProbefor	5’-GTGGTGGACAGTCACAGGTC-3’
Sox2otTargetProberev	5’-GTCAAGGCTTATGGGAATCG-3’
**qPCR**	
mSox2OTbcforQ	5’-GACGCTGATGGGAGAGACTGGTC-3’
mSox2OTbcrevQ	5’-CTCGTCAGCCCAAGCTGGATC-3’
mSox1UTRforQ	5’-CCGAGCGCCAGGTGACATC-3’
mSox1UTRrevQ	5’-GTTGGCATCGCCTCGCTGG-3’
mSox2UTRforQ	5’-GTTCTAGTGGTACGTTAGGCGCTTC-3’
mSox2UTRrevQ	5’-GGACATTTGATTGCCATGTTTATCTCG-3’
bActinforQ	5’-TCGGTGAGCAGCACAGGGTG-3’
bActinrevQ	5’-CGCCCTAGGCACCAGGGTGTG-3’
mMyl6revQ	5’-CTCGGCGTTGGTAGGGTTCTG-3’
mMyl6forQ	5’-CAAGGAGGCTTTCCAGCTGTTTG-3’
mTubb3forQ	5’-TGGACAGTGTTCGGTCTGG-‘3
mTubb3revQ	5’-CCTCCGTATAGTGCCCTTTGG-‘3
mBra(T)revQ’	5’-GTCCAGCAAGAAAGAGTACATGGC-3’
mBra(T)forQ	5’-GCTTCAAGGAGCTAACTAACGAG-3’
Neat1forQ	5’-ACTGGGTGGTTGAGTGGCAA-3
Neat1revQ	5’-TCTGAGCAGGGCTGTGAACC-3
18SforQ	5’-CTCAACACGGGAAACCTCAC-3’
18SrevQ	5’-CGCTCCACCAACTAAGAACG-3’
**ChIP**	
Sox2otH3K4me3for	5’- GAGGGTGTGTTTATTCCTGCTCCAG-3’
Sox2otH3K4me3rev	5’- GCAACGGCTCCTGAATGTCCATC-3’
mMyl6revQ	5’- CTCGGCGTTGGTAGGGTTCTG-3’
mMyl6forQ	5’- CAAGGAGGCTTTCCAGCTGTTTG-3’
**3C**	
SRR1	5’-TTCGCCACCGTTGTCCACATC-3’
IntergenicNegF	5’-GCAGTCTGTGCGTACCATTCTG-3’
SCR-P300boxDD	5’-TCCAGGCTAGAGGACAGTTTGTATAAT-3’
Dppa2intV	5 AACGACTTGATTGTTCTTCCAGGT’-3
Dppa2baitZ	5’-CCTCTGACTAAGGTACCCACACT-3’

Supplementary Methods Information

ChIP-seq data processing as performed and described by Mikkelsen et al. 2007

(*Genome-wide maps of chromatin state in pluripotent and lineage-committed cells. Nature. 2007 448:553-60*)

Sequence reads from each IP experiment were aligned to the mouse reference genome (mm8) using the ARACHNE computational pipeline. First, a table was pre-computed to associate all possible 12-mers with all of their occurrences in the genome. For each ChIP-Seq read (forward and reverse complement orientation), each potential start point was then found and the number of mismatches in the corresponding gap-free alignment was computed. All uniquely aligned reads (defined as the second to best alignment having >2 mismatches more than the best alignment, and the total mismatch count being <=6) were kept. If multiple reads aligned to the same starting position, only one were kept. Fragment densities were computed by counting the number of reads (extended to 300 bp) overlapping each position in the genome (at 25 bp resolution). Non-unique positions in the reference genome were pre-computed by aligning every 27-mer in the genome to the whole genome and masking positions that did not meet the uniqueness criteria defined above.
